# Application of an E-Tongue to the Analysis of Monovarietal and Blends of White Wines

**DOI:** 10.3390/s110504840

**Published:** 2011-05-03

**Authors:** Manuel Gutiérrez, Andreu Llobera, Andrey Ipatov, Jordi Vila-Planas, Santiago Mínguez, Stefanie Demming, Stephanus Büttgenbach, Fina Capdevila, Carme Domingo, Cecilia Jiménez-Jorquera

**Affiliations:** 1 Instituto de Microelectrónica de Barcelona (IMB-CNM), CSIC. Campus UAB, 08193, Bellaterra, Spain; E-Mails: manuel.gutierrez@imb-cnm.csic.es (M.G.); andreu.llobera@imb-cnm.csic.es (A.L.); andrey.ipatov@imb-cnm.csic.es (A.I.); jordi.vila@imb-cnm.csic.es (J.V.-P.); 2 Estació de Viticultura i Enologia, Institut Català de la Vinya i el Vi (INCAVI). Plaça Àgora, 2-3, 08720, Vilafranca del Penedès, Spain; E-Mails: santiago.minguez@gencat.cat (S.M.); fcapdevila@gencat.cat (F.C.); carme.domingo@gencat.cat (C.D.); 3 Institut für Mikrotechnik, Technische Universität Braunschweig, Alte Salzdahlumer Straβe 203, 38124, Braunschweig, Germany; E-Mails: s.demming@tu-bs.de (S.D.); s.buettgenbach@tu-bs.de (S.B.)

**Keywords:** hybrid electronic tongue, electrochemical microsensors, photonic lab on a chip, wine analysis, multivariate chemometric tools

## Abstract

This work presents a multiparametric system capable of characterizing and classifying white wines according to the grape variety and geographical origin. Besides, it quantifies specific parameters of interest for quality control in wine. The system, known as a hybrid electronic tongue, consists of an array of electrochemical microsensors—six ISFET based sensors, a conductivity sensor, a redox potential sensor and two amperometric electrodes, a gold microelectrode and a microelectrode for sensing electrochemical oxygen demand—and a miniaturized optofluidic system. The test sample set comprised eighteen Catalan monovarietal white wines from four different grape varieties, two Croatian monovarietal white wines and seven bi- and trivarietal mixtures prepared from the Catalan varieties. Different chemometric tools were used to characterize (*i.e.*, Principal Component Analysis), classify (*i.e.*, Soft Independent Modeling Class Analogy) and quantify (*i.e.*, Partial-Least Squares) some parameters of interest. The results demonstrate the usefulness of the multisensor system for analysis of wine.

## 1. Introduction

Electronic tongues have been applied to many different fields in the last decades. However, it is in the food quality control and safety where the applicability of these biomimetic systems has been explored more [1]. Wine, which is a high added value product, is not an exception and to date one can find more than 60 papers in the bibliography describing different electronic tongues for classifying wines [2], quantifying parameters of interest for the wine-making industry [3] and even correlating with sensorial descriptors provided by an expert panel [4]. However, these electronic tongue systems are mainly optimized for the analysis of monovarietal wine samples [5,6]. The characterization, identification and even quantification of grape varieties in must and wine mixtures are of great interest to wine-makers [7]. Until now, most papers reporting the resolution of mixtures are based on the analysis of the residual grape DNA by using microsatellite markers [7–9]. This technique is highly reliable, but it requires several experimental steps (sample preparation, DNA extraction, microsatellite amplification and gel electrophoresis) and skilled personnel to carry out. Thus, it is interesting for winemakers to develop a simple and rapid method to characterize a must or wine blend.

Recently, a review has covered the research done in the field of electronic and bioelectronic tongues for the analysis of wine samples [10]. However, one special point that was not covered enough in this review is the data fusion of various measurement techniques (potentiometry, amperometry, conductance, spectrophotometry, gas sensing). These systems are called hybrid electronic tongues because they merge variables of different nature. This approach was already reported in the end of nineties by Toko and coworkers as a powerful way to improve the performance of sensor technologies to the analysis of wines [11,12]. In this later work the combination of a taste sensor array [13] and a smell sensor array was applied to discriminate wines. This fact was reinforced by the formulation of the taste sense as the sum of taste, smell, texture, color, sound and temperature [14]. Since then, just four hybrid electronic tongues for wine have appeared in the bibliography, including those that also merge optical variables [4,15–17].

In our previous work [17], the application of a hybrid multiparametric system based on electrochemical and colorimetric measurements was presented. That was the first time in which a miniaturized optofluidic system was used as part of an electronic tongue for the analysis of white and red wines. Now, we present the next steps in which some improvements are incorporated to the system. First of all, the colorimetric analysis is performed using a multiple internal reflection (MIR) photonic chip fabricated by soft-lithography [18]. The main advantage of the MIR configuration is an increase in sensibility in comparison with the hollow prism configuration used in [17], thanks to the use of a longer optical path. This is especially important in the analysis of white wine, given the small absorbance values of this type of sample. Secondly, a flow cell has been fabricated in order to automate part of the measurements, concretely the potentiometric ones using Ion-Sensitive Field Effect Transistors (ISFETs). Besides, many different chemometric tools have been used in order to obtain the best models: Principal Component Analysis (PCA) combined with different strategies for characterization, Soft Independent Modeling Class Analogy (SIMCA) for classification and Partial-Least Squares (PLS) for quantification. Also in this work, the sample set is formed by 20 monovarietal white samples from six grape varieties. This group of samples was complemented with seven bi- and trivarietal wine mixtures. The analysis of these samples with the multiparametric system formed by the array of electrochemical microsensors and a colorimetric optofluidic system has demonstrated the feasibility of the system to classify white wines according to the grape varieties and to analyse some specific parameters.

## 2. Experimental Section

### 2.1. Reagents and Solutions

All reagents used were of high purity, analytical grade or equivalent. All solutions were prepared with de-ionized water. For ISFET calibration, solutions with ionic salts with concentrations between 10^−2^ and 10^−4^ M were prepared. In the case of those sensitive to cations (Na^+^, K^+^ and Ca^2+^), the corresponding chloride salts were considered. For the Cl^−^ and NO_3_^−^ ions, solutions of NaCl and KNO_3_, respectively, were prepared. A solution containing 0.1 M KNO_3_ was used to activate the amperometric gold (Au) sensor. In order to calibrate the conductivity sensor, two different standard solutions from Crison (Barcelona, Spain), with nominal values of 1,413 and 147 μS/cm, were utilized. Two standard redox solutions from Panreac (Barcelona, Spain), with values of 468 mV and 220 mV, were used to calibrate the oxido-reduction potential (ORP) sensor. Finally, for the electrochemical oxygen demand (EOD) electrode, a solution containing 0.1 M NaOH and a glucose stock solution of 25 g/L were used. For the ISFET measurements, a reference solution containing an average concentration of the main species present in wine was prepared. The composition of this solution has been reported elsewhere [17].

### 2.2. Wine Samples and Mixtures

The set of samples consisted of 18 monovarietal (100%) white wines based on the following grape varieties: Macabeu (samples 09/07, 10/07, 11/07, 12/07 and 17/07), Parellada (samples 77/07, 78/07, 79/07, 80/07 and 81/07), Chardonnay (samples 01/07, 02/07, 03/07 and 04/07) and Xarel·lo (samples 43/07, 44/07, 45/07 and 63/07), all provided by the Catalan Institute of Vineyard and Wine (INCAVI). The index “07” indicates that the samples correspond to the 2007 vintage. All the samples have the same geographical origin (Penedes), except the Macabeu samples 11/07 and 12/07, which came from the Garraf zone. The winemaking process for all these samples is the same. It begins with the reception of the grapes in the winery. Processes of crushing, pressing and the addition of sulfites to the grape juice are performed successively. Then, a first clarification in order to eliminate the suspended solids and the alcoholic fermentation that transform the grape juice into wine are done. Once the wine is obtained, a second stage of clarification followed by stabilization and filtration steps and a second addition of sulfites are performed, before bottling the final product.

Samples were provided by INCAVI in 750 mL bottles. Aliquots of 25 mL were collected from each wine sample in bottles protected against light and put into the refrigerator at 4 °C for a maximum of one week. Two hours before the analysis, they were removed from the fridge to equilibrate at room temperature. During the analysis, the aliquots were successively opened and analyzed to prevent the possible changes of composition. Besides, two monovarietal white wines from Croatia based on Grasěvina and Zelenac grape varieties were included in the model and seven different wine mixtures were prepared from the Catalan samples, thus giving bivarietal and trivarietal wines, whose composition is shown in Table 1. These blends are typical for still white wines and sparkling wines by considering the oenological properties of these varieties: the Macabeu one gives light and fruity wines, the Xarel.lo gives wines with body and acidity and the Parellada, light and aromatic wines.

Certain chemical parameters in these wines were determined by the INCAVI using standard methods. These methods are dictated by the European Union [19] and are well-established by the INCAVI laboratories. The routine protocol consists on one measurement for sample, without repetitions. Some of parameters, such as the volumetric alcoholic degree (VAD), the pH, the magnesium and calcium ions, are analyzed to evaluate the technological efficiency of the process, while others such as the total acidity are to meet legal limits. Some optical parameters were also determined by the INCAVI, such as the color of the wines by means of CIELab coordinates [20], the intensity of color and the tonality. These last two parameters correspond to the sum of absorbances at 620, 520 and 420 nm, and the ratio between the absorbance at 420 nm and 520 nm [19], respectively.

### 2.3. Hybrid Electronic Tongue

A set of six ISFET sensors were fabricated using standard microelectronic technology [21]. One ISFET was used for measuring pH and the rest were modified with polymeric membranes sensitive to Na^+^, K^+^, Ca^2+^, Cl^−^ and NO_3_^−^ ions. Polymeric membranes were based on photocurable polymers with commercial ionophores from Fluka (Buchs, Switzerland). The ionophores used in each case were: 4-*tert*-butylcalix[4]arenetetraacetic acid tetraethyl ester (Ionophore X) for Na^+^, valinomycin (Ionophore I) for K^+^, *N,N,N′,N′*-tetracyclohexyl-3-oxapentanediamide (Ionophore II, ETH 129) for Ca^2+^, tridodecylmethylammonium chloride for Cl^−^ and tetraoctylammonium nitrate for NO_3_^−^. All these ionophores are selective to the principal ion, but are not specific and they present a certain degree of cross-response to other ions in solution. Membrane composition, preparation and characterization has been presented elsewhere [22,23]. An Orion 90-02-00 double junction Ag/AgCl reference electrode (Thermo Electron, Waltham, MA, USA) with 0.1 M CH_3_COOLi solution in its outer chamber was employed for all the potentiometric measurements. These were performed with the aid of a laboratory constructed data-acquisition system, which was connected to a computer through the RS-232 board. The readings were done employing specially designed software programmed with VisualBasic (Microsoft, Seattle, WA, USA).

**Figure 1.**
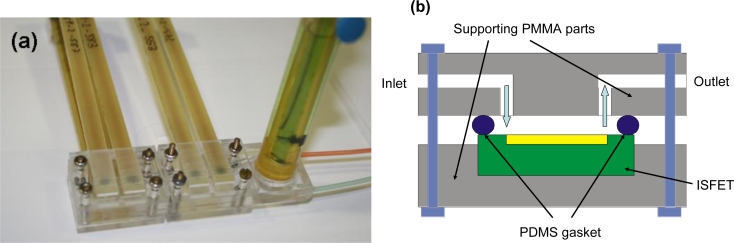
(**a**) Picture of the flow cell employed for the ISFETs measurements and (**b**) a detailed scheme of an ISFET cell.

A flow cell (presented in Figure 1) to incorporate the ISFETs was made of a polymethyl-methacrylate (PMMA) plate with the help of a micro-milling machine (Stepfour GmbH, Salzburg, Austria). Different milled parts of the PMMA were glued together with methacrylic acid. For tight contact between the flow cell and the individual ISFET, a PDMS (polydimethylsiloxane) gasket formed in a special mould was used. To ensure that all the ISFET cells have no leakage three separate covers were made. The flow cell channel has 0.5 mm width and 0.5 mm high. Each ISFET has a 4 mm × 2 mm × 0.5 mm cell with a wall jet inlet configuration. The outlet from this cell is connected with inlet of the next ISFET, so all six ISFETs were placed sequentially. Standard 0.8 mm inner diameter tubing for flow injection analysis was connected to the inlet and outlet of the cell. The reference electrode (Orion) was mounted in a special flow-through cell connected to the output of the ISFET cell.

Sensors based on a Pt 4-electrode configuration were employed as conductivity sensor and ORP sensor. Their fabrication and characterization are reported elsewhere [24]. For signal conditioning and control data acquisition, a versatile and portable system developed at the IMB was used. This system was connected to the PC through the USB link. The control programs were based on LabView graphic language (National Instruments, Austin, TX, USA).

Two different amperometic microsensors were employed: a conventional Au microelectrode fabricated according to standard photolithographic techniques and a composite planar electrode (CPE) for sensing electrochemical oxygen demand developed by our group [25]. In both cases, the amperometric cell contained the working electrode, a Pt commercial electrode as counter electrode (Radiometer, Lyon, France) and a Ag/AgCl/10% (w/v) KNO3 reference electrode (Metrohm 0726 100, Herisau, Switzerland). A μ-Autolab potentiostat/galvanostat (Ecochemie, Utrecht, The Netherlands), using GPES 4.7 software package (General Purpose Electrochemical System) was used for all voltammetric and amperometric measurements.

Optical measurements were done using an MIR configuration fabricated in poly(dimethylsiloxane) (PDMS). The photonic lab on a chip comprises microlenses, self-alignment structures, microfluidic channels and air mirrors for highly sensitive absorbance measurements [18]. The light was emitted from broadband light source (Ocean Optics HL-2000, Redwood Shores, CA, USA). The readout multimode optical fibre was connected to a spectrometer (Ocean Optics HR-4000) with a spectral resolution of 2 nm. Using the Ocean Optics SpectraSuite software, the absorption of the sample from 200 to 1,100 nm wavelengths was recorded.

### 2.4. Methodology

Sample analysis was carried out under batch conditions, except for the ISFET set, which was performed under flow conditions. A complete analysis of a wine sample took around 15 min. No replications of each sample were done following the INCAVI protocol and also to get a rapid analysis and prevent changes of the wine sample. All the sensors that form the array were characterized before the analysis. The response characteristics are reported in [17], including the precision for the measurement of one wine sample.

For the ISFET set, the output signals corresponded to the relative measurements of each sensor with respect to the reference solution, which was checked periodically. This is a common strategy to correct the possible drift of the sensors.

The 4-electrode sensor was firstly chemically cleaned successively with ethanol 96%, H_2_SO_4_ 6.0 M and de-ionized water. Calibration of the conductivity sensor was carried out using two standard solutions (1,413 and 147 μS/cm). For ORP sensor evaluation, a test with standard redox solutions of 220 and 468 mV (at 25 °C) *versus* the Ag/AgCl reference electrode was performed. Once the good behaviour of the sensors was confirmed, they were immersed in the wine sample and the signals were recorded every 30 seconds during 3 minutes.

The Au microelectrode was firstly chemically cleaned as before, followed by an electrochemical activation carried out in 0.1 M KNO_3_ where the electrode was cycled from +0.8 to −2.2 V for at least 20 times. Then the electrode was immersed in the sample under studied and two cyclic voltammograms (CV) from +1.6 to −0.5 V at a scan rate of 0.1 V/s were run: the first one to stabilize the signal and the second one to obtain the information. Finally, the electrode was activated after measuring each wine samples by running five CV in 0.1 M KNO_3_.

Regarding the electrochemical oxygen demand sensor (EOD) based on a CPE, previous studies in our laboratories have demonstrated the response of this electrode towards glucose and some other sugars. For this reason, analyses were performed using glucose as internal standard. Using the chronoamperometric mode and setting the potential at +600 mV, the CPE was immersed in NaOH 0.1 M. Then 50 μL of glucose 25 g/L was added. When the response became stable, 100 μL of the studied wine was added. In this case, the output signals used to construct the model were the ratio of the wine signal to the glucose signal.

In case of the photonic lab on a chip, measurements were carried out by filling the system with the sample to be analyzed. In order to obtain the spectra in absorbance units, de-ionized (DI) water was used as reference. Measurements were taken prior and after each wine to determine signal drifts due to non-specific adsorption at the walls. Throughout the experiments, the DI water reached the same value between the experimental errors, confirming the reversibility of the photonic lab on a chip. Finally, for statistical purposes, for each wine type, the average of 10 consecutive scans was considered.

### 2.5. Data Management

Obtained data were treated using different multivariate methods. Principal Component Analysis (PCA) was used as the first technique to evaluate the characterization power of the system for monovarietal and mixture samples. The Soft Independent Modeling Class Analogy (SIMCA) method was also utilized to achieve a good classification model for the wine mixtures. The Partial Least Squares (PLS) regression was employed to perform the quantification of different parameters of the samples [26]. In this work, the PLS-1 algorithm was used in order to obtain more accurate predictions.

For all these methods, the original values were previously autoscaled—all the variables were centered and set to a standard deviation equal to 1—to avoid variables from having a different influence on the model. Besides, all the obtained models were centered. For the PCA and SIMCA analyses, the cross-validation technique was used in order to ensure the performance of the generated model. In typical cross-validation, the training and validation sets of samples cross-over in successive iterations such that each sample has a chance of being validated against. Contrary, the test-set validation technique was used for the PLS regressions. In this case, a fixed calibration and prediction sets of samples are chosen. To control all these parameters and to perform the analyses, the Unscrambler v.9.1 informatics package (CAMO ASA, Oslo, Norway) was used.

## 3. Results and Discussion

### 3.1. Input Variables for the Models

Once all the samples were passed through the sensors, a data matrix was constructed with the different variables to be used as the input of the chemometric tool. These variables are fixed for ISFET sensors, conductivity, ORP and EOD sensors but the value of current and the absorbance depends on the specific response of the sensor to the samples. For this reason the election of the variables is an important step in a multivariable analysis. In other words, the goodness of the models generated by the algorithms depends essentially on the success of this choice. In the present study, the input data was composed by 16 variables as shown in Table 2.

**Figure 2.**
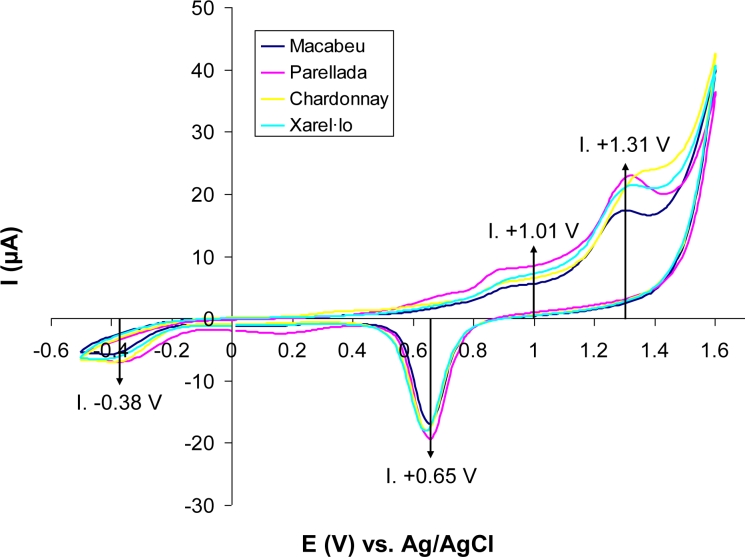
Cyclic voltammograms obtained with the Au microelectrode for four Catalan wines, one of each grape variety. Intensities for the indicated peaks were used as variables for the model.

The input data of the six ISFETs correspond to the relative signal in mV of each ISFET with respect to the reference solution. It is also the case of the conductivity, ORP and EOD sensors, which give an absolute signal as a result. For the measurements with the Au microelectrode, cyclic voltammograms (CV) were carried out. In Figure 2, the obtained CVs for four Catalan wines, one of each grape variety, are represented. The results show two redox peaks corresponding to the oxidation (+1.31 V) and reduction of Au (+0.65 V) from the electrode. Besides, two smaller peaks are observed at +1.01 V and at −0.38 V, which maybe correspond to the polyphenols content since their antioxidant capacity is well-known [27]. Therefore, the intensity of these four peaks was used as new variables. Also for the optofluidic system a spectrum of absorbance as function of the wavelength is obtained. In order to choose the variables, we applied the EU method that defines the color of wine at absorbance of 420, 520 and 620 nm [19]. With these variables, we obtained the maximum information about the wine samples.

**Figure 3.**
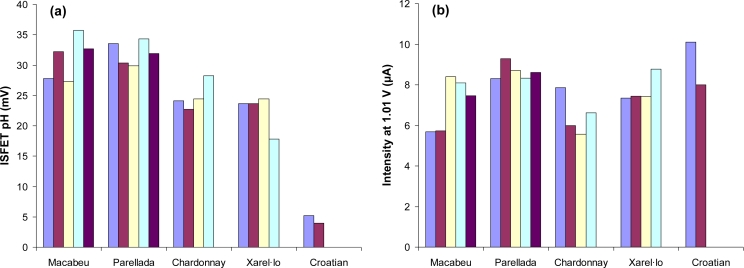

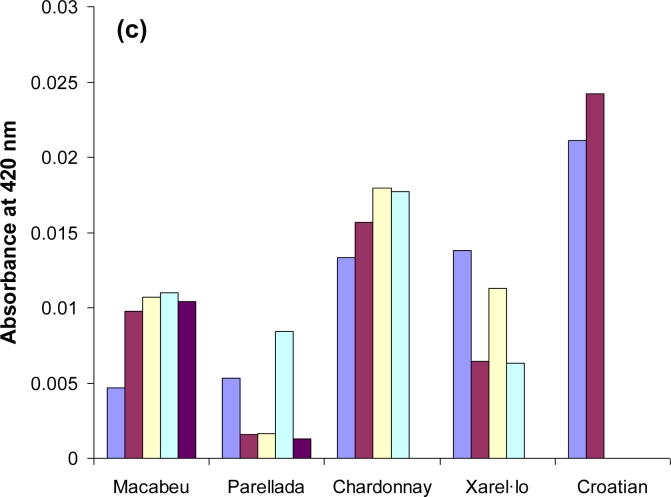
Response of sensors for the 20 monovarietal wines: (**a**) pH ISFET, (**b**) Au microelectrode at +1.01 V and (**c**) optofluidic system at 420 nm.

As an example of the values obtained by the different sensors, the response of three representative variables for the 20 monovarietal wines is represented in Figure 3.

For the pH ISFET [Figure 3(a)] similar values can be observed for the Macabeu and Parellada samples, and also comparable values for Chardonnay and Xarel·lo samples. However, values for the Croatian wines are significantly different due to the higher pH of these wines [28]. Figure 3(b) shows the intensity of current at +1.01 V using the gold microelectrode. The analysis of this graph shows homogeneous values for the Perallada group and more variability within the rest of grape varieties. For example, this is the case of the Macabeu samples, which form two groups with three and two samples, respectively. Finally, the results for the absorbance at 420 nm using the optofluidic system [Figure 3(c)] show again the differences between the Croatian wines and the rest. Besides, it can be observed some variability between the Catalan grape varieties with the highest values of absorbance for the Chardonnay group and the lowest for the Parellada group.

### 3.2. Characterization of the Monovarietal Samples

**Figure 4.**
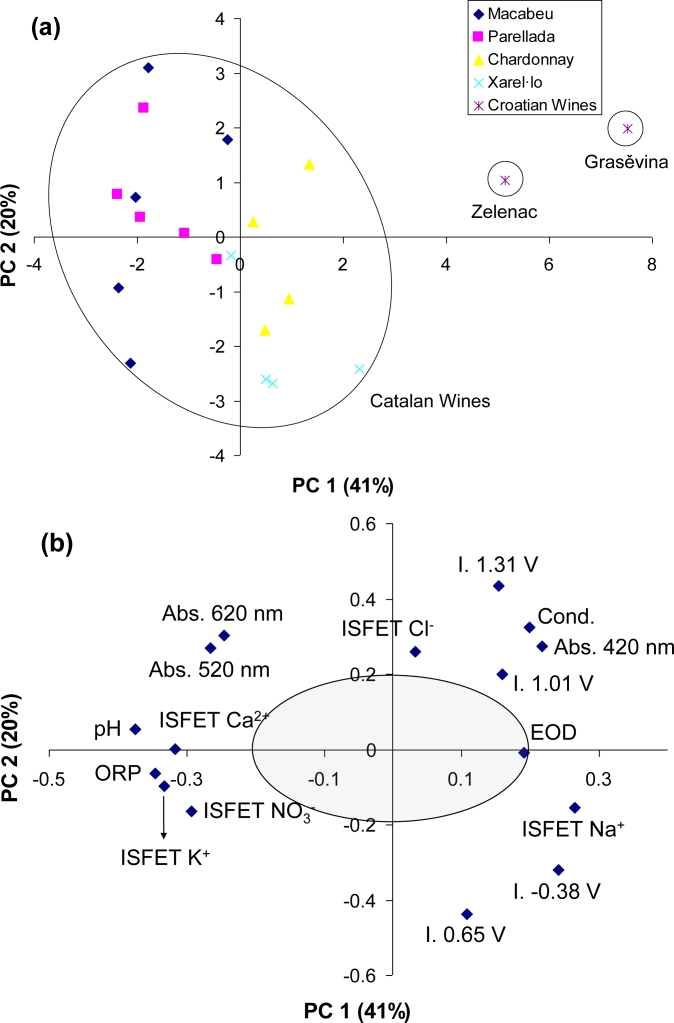
PCA results for the 20 monovarietal wine samples: (**a**) scores plot and (**b**) loadings plot with a grey circumference which corresponds with the zone of low significance.

In order to prove the capability of the system to differentiate monovarietal samples from different geographical origin, a PCA was performed using the 20 monovarietal wines: the 18 Catalan samples and the two Croatian samples. These Croatian wines are based on two characteristic grape varieties from the Slavonia zone: Grasěvina White and Zelenac. In Figure 4(a) is represented the scores plot with the two first principal components (PC), which explain 61% of the total variance. In this model, the first clear conclusion is that the Croatian wines are outliers of the population; they are completely different from the Catalan white wines. Grasěvina and Zelenac grape varieties are very distinguishable even between each other. On the contrary, the Catalan wines are intermixed forming a unique group. However, a slight separation is glimpsed between the Macabeu and Parellada samples placed in the negative PC1 axis and the Chardonnay and the Xarel·lo samples placed in the positive PC1 axis.

It is interesting to analyze which is the weight of each original variable in the constructed model. We can observe in Figure 4(b) that all the used variables are located far from the origin (0, 0), except the EOD sensor that is situated in the border of the low-significant zone. The significance of the variables is directly related with the distance to the origin: the more away, the more importance in the model. Therefore, the pH ISFET has the biggest weight in the PC 1, which explains the 41% of the variance. The ORP sensor and the ISFETs for K^+^ and Ca^2+^ have also importance in the first PC, while the PC 2 is composed basically by two variables of the Au microelectrode (intensities at +1.31 and +0.65 V). However, the three optical variables have a discrete contribution both to the first component (between 0.21 and 0.27) and to the second component (between 0.27 and 0.30). In other words, the electrochemical variables have a higher weight for the monovarietal white wine classification than the optical ones, and could be removed of this first model.

**Figure 5.**
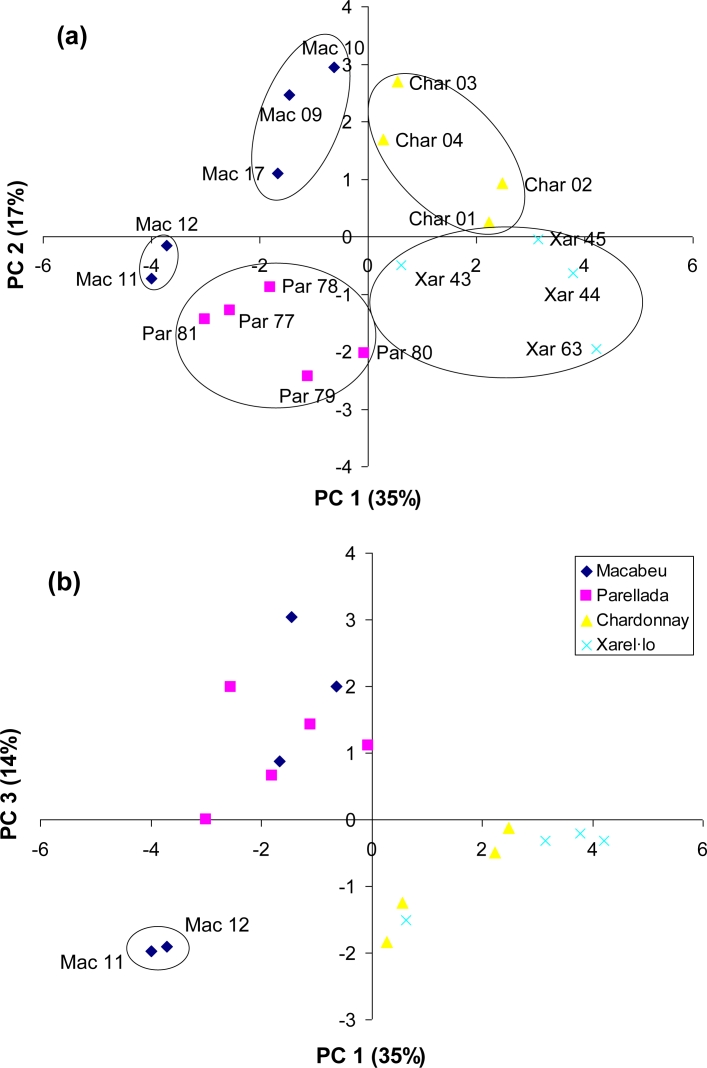
PCA scores plots for the monovarietal Catalan wines using the values obtained with the hybrid system: (**a**) PC 1 (35%) *vs.* PC 2 (17%) and (**b**) PC 1 (35%) *vs.* PC 3 (14%).

The next study performed with this system was the characterization of the 18 Catalan wine samples. A new PCA was performed using the raw data obtained from the selected variables. In Figure 5(a) the two first principal components, which explain 52% of the total variance, are represented. In this scores plot, the four varieties are distributed in the four quadrants: Macabeu, Parellada, Chardonnay and Xarel·lo. However, the two Macabeu samples from the Garraf zone (Mac 12/07 and Mac 11/07) are separated of those from the Penedes zone and they form a new distinguished group. These two Macabeu wines correspond to the samples with lower values for the intensity of current at +1.01 V [Figure 3(b)]. Besides, some variability inside the Parellada, Chardonnay and Xarel·lo groups can be observed. As can be observed in Figure 5(b), the PC 3 of this model explains 14% of the variation of the Catalan wines and clearly separates the two Macabeu samples from Garraf of the rest of the Macabeu samples from Penedès. On the other hand, this third component does not discriminate between the Macabeu and Parellada wines, nor between the Chardonnay and Xarel.lo wines. In the loadings plot of this new model (data non-shown), the most important variables are again the electrochemical ones: pH ISFET and the variables of the Au microelectrode (intensities at +1.01, +0.65 and −0.38 V). However, the optical variables have more weight in comparison with the previous model, especially the absorbance at 620 nm that has a contribution of 0.32 in the first PC.

**Figure 6.**
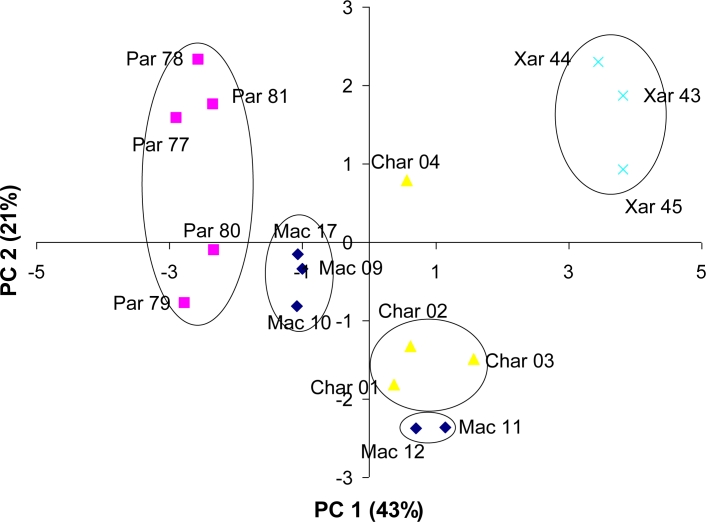
PCA scores plot for the monovarietal Catalan wines using the chemical and optical parameters determined by standard methods of analysis.

In order to compare the capability of our system to differentiate Catalan wine samples with the data from standard methods, a PCA using the chemical and optical data provided by the INCAVI was performed. The obtained scores plot along the first two components is shown in Figure 6. The white wine samples are aligned on the plot according to the grape variety along the first principal component, which explains 43% of the variance. Besides, the second component increases the dispersion of the Parellada samples, which seem to form two groups, as we observed in the PCA using the hybrid system [Figure 5(a)]. This PC 2 distinguishes Char 04/07 from the other Chardonnay samples and separates better the Xarel·lo group from the rest. Besides, the two Macabeu samples from the Garraf zone are also separated of the rest of the Macabeu group. In this case, these two samples are intermixed with the Chardonnay group. This fact confirms the differences within the Macabeu group depending on the geographical origin that are detected not only by the hybrid electronic tongue but also by data from standard methods of analysis. However, it is noticeable the highest power of wine samples classification of our system.

### 3.3. Characterization of the Bi- and Trivarietal Samples

**Figure 7.**
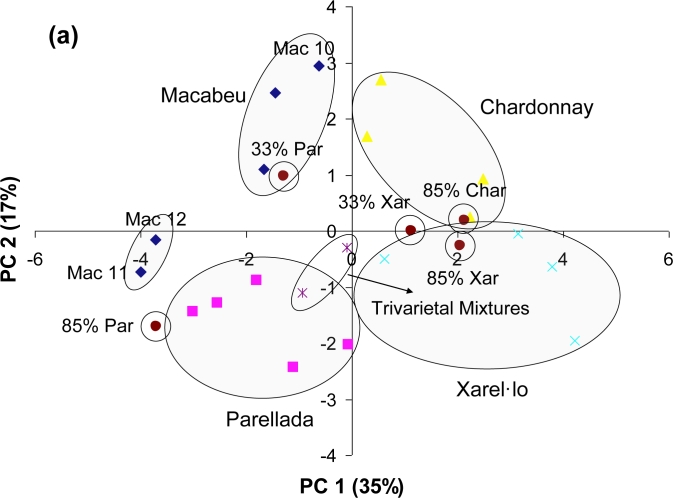

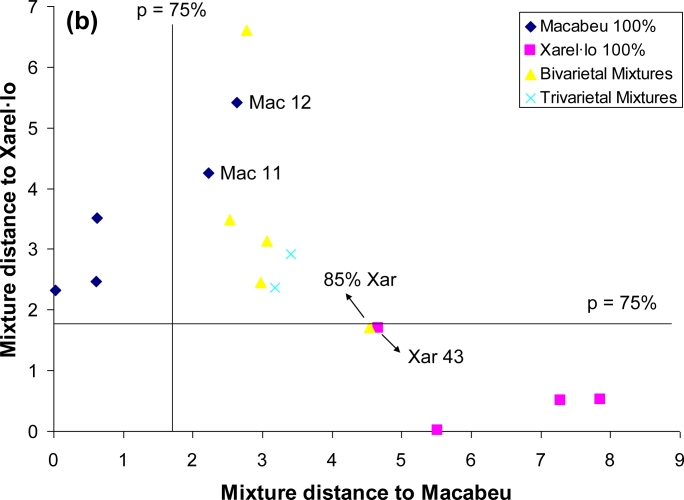
(**a**) Interpolation of the multivarietal mixtures in the PCA model; (**b**) Coomans diagram for the Macabeu and Xarel·lo models with a probability of 75%.

Once the model using raw data from the sensor variables was constructed, the values obtained for the seven mixtures were interpolated in the PCA. The situation of these samples inside the model is shown in Figure 7(a). As can be observed, trivarietal mixtures, which are formed by 33.33% of each variety, tend to be near the origin (0,0). Bivarietal samples with 85% of one variety are inside the group of the major variety, being almost impossible to distinguish them from the monovarietal wines. However, the mixtures that content 66.66% Mac 10/07 and 33.33% Parellada or Xarel·lo seem to be halfway between the two groups, as if there is a gradation in terms of percentage of each variety. In order to study in more detail the feasibility of the hybrid electronic tongue to distinguish between the monovarietal (100%) wines and the mixtures, the SIMCA classification method was applied. In Figure 7(b), the Coomans diagram (probability of 75%) obtained for the classification of the seven wine mixtures among the Macabeu and Xarel·lo models is depicted. These two groups are studied because they are well characterized by the system. In the x-axis of the Coomans diagram, the mixture distance to the Macabeu model is shown. Thus, mixtures that are placed between 0 and 1.75 on the x-axis belong to the Macabeu model. On the y-axis, the mixture distance to the Xarel·lo model is shown. Hence, mixtures that are placed between 0 and 1.75 on the y-axis belong to this group. Mixtures that are placed in the area corresponding to x, y > 1.75 do not belong to any of the represented models and can be distinguished from the monovarietal wine samples. Contrary, mixtures that are located in the area x, y < 1.75 of the plot belong to both represented models. As can be observed, the monovarietal Macabeu wines (blue diamonds) are located inside their model and are well separated of the rest of wine mixtures. It is important to say that all these mixtures have at least 15% of a Macabeu wine. However, the two Macabeu samples from the Garraf zone (Mac 11 and 12) are located outside the Macabeu group, which is formed by the Penedes samples, confirming that the hybrid system is capable of differentiating between them. At the same time, the monovarietal Xarel·lo wines (pink squares) are also inside their own model and are well distinguished of the rest of wine mixtures, in general. The case of the Xar 43/07 and the mixture with 85% Xarel·lo is remarkable in that, with a y value of 1.70 and 1.71 respectively, are just in the border of the Xarel·lo model. It is important to say that although the probability of the classification model is 75%, this is a first step to evaluate the feasibility of the system in the resolution of wine mixtures.

### 3.4. Quantification of Chemical and Optical Parameters

Next, a PLS was realized in order to assess if the system was able to quantify some quality parameters of the samples already analyzed with standard methods. For the regression, the calibration set was formed by 14 samples: four Macabeu samples (10/07, 11/07, 12/07 and 17/07), four Parellada samples (78/07, 79/07, 80/07 and 81/07), three Chardonnay samples (01/07, 03/07 and 04/07) and three Xarel·lo samples (43/07, 45/07 and 63/07). The rest of the samples were used as the prediction data set. The relative standard errors of the calibration set (RSEC) and the prediction set (RSEP) were calculated in order to compare the precision of the different PLS regressions. The RSEC values were between 1.0 and 5.0% for all the determinations and the RSEP values were between 2.2 and 7.6%, without excluding any value.

The data of optical and chemical parameters for the prediction set obtained with our system and with standard methods are shown in Tables 3 and 4, respectively. The interpolated values are in good agreement with the data obtained using standard methods. The relative errors are below 10% for all the predictions, except for the determination of glycerol in the 44/07 Xar sample. Especially good results are obtained for VAD, pH and magnesium predictions, with errors below 5%. Besides, the inclusion of the three optical variables permits to quantify with a high precision the intensity of color and the tonality of the wine. As can be observed, for these two parameters the errors are in general below 5%.

## 4. Conclusions

This work demonstrates the feasibility of using the proposed hybrid electronic tongue to analyze monovarietal white wines and their bi- and trivarietal mixtures. The high complexity of the wine matrix makes particularly interesting the combination of electrochemical and optical variables in order to obtain the maximum sample information. Then, this obtained data is treated with powerful chemometric tool to extract useful chemical information.

The qualitative results using PCA technique confirm that this system is capable of distinguishing the samples according to the grape origin and even to the geographical origin. Using the SIMCA classification technique, the ability of the system to differentiate between the monovarietal samples and their mixtures has been demonstrated with a probability of 75%. In addition, the application of the PLS technique to the collected data, permits one to quantify some chemical and optical parameters with relative errors smaller than 10% in general, obtaining especially good results for the prediction of VAD, pH, magnesium and tonality. Even though the electrochemical variables seem to be more significant for these analyses, the optical ones cannot be undervalued because they are essential for some applications, like the correct differentiation of the Catalan wine set and the quantification of optical parameters.

In conclusion, good results are obtained both for discrimination and quantification methodologies that confirm the viability of the multisensor system. Ongoing experiments are focused on processing a higher number of samples, from different varieties, vintage years and origin to improve the reliability of the system. Moreover, an important effort will be performed to analyze qualitatively bi- and trivarietal samples and even to quantify the proportion of each grape variety presents in the wine mixture. Besides the integration of sensors in the same substrate in order to obtain a compact system that could be applied to flow measurements will permit more automated and feasible analysis.

## Figures and Tables

**Table 1.** Composition of the seven wine mixtures prepared from the Catalan monovarietal samples.

**Mixture**	**Macabeu**		**Parellada**		**Chardonnay**		**Xarel·lo**	

	**Sample**	**(%)**	**Sample**	**(%)**	**Sample**	**(%)**	**Sample**	**(%)**
1	17/07	33.33	79/07	33.33	-	-	44/07	33.33
2	12/07	15	-	-	01/07	85	-	-
3	12/07	15	77/07	85	-	-	-	-
4	12/07	15	-	-	-	-	43/07	85
5	11/07	33.33	78/07	33.33	-	-	45/07	33.33
6	10/07	66.66	80/07	33.33	-	-	-	-
7	10/07	66.66	-	-	-	-	45/07	33.33

**Table 2.** Variables considered for constructing the models.

**Devices**	**Variables**
ISFETs	pH, Na^+^, K^+^, Ca^2+^, Cl^−^ and NO_3_^−^
4-bars electrode	Conductivity and ORP
Au microelectrode	Current at 1.31 V, 1.01 V, 0.65 V and −0.38 V
Composite microelectrode	EOD
Optofluidic system	Absorbance values at 420, 520 and 620 nm

**Table 3.** Quantification with PLS technique of some optical parameters of white wine samples with the system. Standard method’s data were provided by INCAVI and relative error refers to these data.

**Sample**	**Standard method**	**Multiparametric system**	**Relative error (%)**
Intensity of color			

Macabeu 09/07	0.0540	0.0565	4.67
Parellada 77/07	0.0520	0.0544	4.56
Chardonnay 02/07	0.0700	0.0706	0.87
Xarel·lo 44/07	0.0960	0.103	7.29

Tonality			

Macabeu 09/07	4.777	4.829	1.09
Parellada 77/07	4.777	4.952	3.66
Chardonnay 02/07	5.272	5.403	2.48
Xarel·lo 44/07	3.000	2.873	−4.23

**Table 4.** Quantification with PLS technique of some chemical parameters of white wine samples with the system. Standard method’s data were provided by INCAVI and relative error refers to these data.

**Sample**	**Standard method**	**Multiparametric system**	**Relative error (%)**
VAD (%)			

Macabeu 09/07	9.59	9.86	2.82
Parellada 77/07	9.37	9.38	0.07
Chardonnay 02/07	11.90	12.30	3.32
Xarel·lo 44/07	13.86	13.77	−0.62

Total acidity (g/L)			

Macabeu 09/07	5.9	5.7	−3.7
Parellada 77/07	7.7	8.4	9.5
Chardonnay 02/07	8.0	7.8	−2.7
Xarel·lo 44/07	5.7	5.8	1.0

pH			

Macabeu 09/07	3.03	3.01	−0.79
Parellada 77/07	2.93	2.83	−3.55
Chardonnay 02/07	3.10	3.14	1.16
Xarel·lo 44/07	3.34	3.18	−4.76

Calcium (mg/L)			

Macabeu 09/07	71	70	−0.7
Parellada 77/07	69	72	5.1
Chardonnay 02/07	59	62	5.7
Xarel·lo 44/07	43	45	4.0

Magnesium (mg/L)			

Macabeu 09/07	48	46	−4.9
Parellada 77/07	43	43	−0.1
Chardonnay 02/07	65	64	−2.2
Xarel·lo 44/07	46	45	−2.8

Glycerol (g/L)			

Macabeu 09/07	6.1	6.5	6.0
Parellada 77/07	4.8	4.9	2.0
Chardonnay 02/07	7.6	7.3	−4.3
Xarel·lo 44/07	6.3	7.1	12.3
